# Endometriosis fertility index score maybe more accurate for predicting the outcomes of *in vitro* fertilisation than r-AFS classification in women with endometriosis

**DOI:** 10.1186/1477-7827-11-112

**Published:** 2013-12-11

**Authors:** Wenjun Wang, Ruiqi Li, Tingfeng Fang, Lili Huang, Nengyong Ouyang, Liangan Wang, Qingxue Zhang, Dongzi Yang

**Affiliations:** 1 Department of Obstetrics & Gynecology, Reproductive medicine centre, Sun Yat-Sen Memorial Hospital, Sun Yat-Sen University, No.107 Yanjiang Xi Road, Guangzhou 510120, P. R. China; 2 New Hope Fertility Center, Av.De Praia Grande No.409, China Law Building, 3 Andar BC, Macau, P. R. China; 3 Endocrinology division, Department of Obstetrics & Gynecology, Sun Yat-Sen Memorial Hospital, Sun Yat-Sen University, No.107 Yanjiang Xi Road, Guangzhou 510120, P. R. China

**Keywords:** Endometriosis fertility index (EFI), Revised American Fertility Society (r-AFS) classification, Endometriosis, In vitro fertilization (IVF), Clinical pregnancy rate

## Abstract

**Background:**

Endometriosis is a common disease. The most widely used staging system of endometriosis is the revised American Fertility Society classification (r-AFS classification) which has limited predictive ability for pregnancy after surgery. The endometriosis fertility index (EFI) is used to predict fecundity after endometriosis surgery. This diagnostic accuracy study was designed to compare the predictive value of the EFI with that of the r-AFS classification for IVF outcomes in patients with endometriosis.

**Methods:**

199 women with endometriosis receiving IVF treatment after surgery were analysis. The EFI score and r-AFS classification in their ability to predict these IVF outcomes were compared in the same population. ROC curves were used to analyse the predictive values of the EFI and r-AFS indices for clinical pregnancy, and their accuracies were evaluated by sensitivity, specificity, and the Youden’s index.

**Results:**

The Area Under the Curve (AUC) of the EFI score (AUC = 0.641, Standard Error(SE) = 0.039, P = 0.001, 95% CI = 0.564-0.717, cut-off score = 6) was significantly larger than that of the r-AFS classification (AUC = 0.445, SE = 0.041, P = 0.184, and 95% CI = 0.364-0.526). The antral follicle count, oestradiol level on day of hCG, number of oocytes retrieved, number of oocytes fertilised, and number of cleaved embryos in the greater than or equal to 6 EFI score group was greater than that of the lower than or equal to 5 EFI score group, and the dose of gonadotropin of the greater than or equal to 6 EFI score group were less than that in the lower than or equal to 5 EFI score group. Implantation rate, clinical pregnancy rate, and cumulative pregnancy rate in the greater than or equal to 6 EFI score group were higher than in the lower than or equal to 5 EFI score group.

**Conclusions:**

It suggests that the EFI has more predictive power for IVF outcomes in endometriosis patients than the r-AFS classification. The clinical pregnancy rate was higher in patients with EFI greater than or equal to 6 score than with EFI lower than or equal to 5 score.

## Background

Endometriosis is a common disease that occurs in 6 to 10% of reproductive-age women [1,2]. Approximately 25 to 50% of infertile women have endometriosis, and 30 to 50% of women with endometriosis are infertile [3]. Today, the most widely used staging system of endometriosis is the revised American Fertility Society classification (r-AFS classification) [4,5]. The r-AFS classification is used to predict the recurrence potential of endometriosis after surgery. However, it has limited predictive ability for pregnancy after surgery. Adamson and Pasta suggested that the r-AFS classification depends mainly on morphological descriptions [6], whereas Vercellini et al. observed no association between the endometriosis stage or lesion type and lesion site and the cumulative probability of pregnancy [7].

The endometriosis fertility index (EFI), proposed by Adamson and Pasta in 2010, is used to predict fecundity after endometriosis surgery [8]. In addition to providing a detailed score to the appendix (fallopian tubes, fimbriae of fallopian tubes, ovaries) by calculating the least-function scores, the EFI also combines conception-related factors such as age, duration of infertility, and gravidity history. The EFI contains all of the components of the r-AFS stage score, but the r-AFS score includes only 20% of the EFI score. However, the EFI does not consider whether the patient has received *in vitro* fertilisation (IVF) treatment after endometriosis surgery [9]. Until now, there is no report on the evaluation of IVF outcomes and embryo quality by the EFI score after endometriosis surgery, and no report was on the comparison of the EFI score with the r-AFS classification in the prediction of IVF outcome. This study aimed to compare the predictive value of EFI score with that of r-AFS classification in the same population of women with endometriosis who had received IVF treatment. We performed a retrospective analysis of the case histories, operative records, and the available information on embryo quality, implantation rate, and pregnancy rate of infertile women who had received IVF treatment after endometriosis surgery.

## Methods

### Study population and design

The study was conducted after receiving Institutional Review Board approval. Patients were provided with counseling, and signed consents were obtained.

A total of 199 women of reproductive age who had endometriosis and received their first cycle of IVF treatment between January 2008 and July 2012 were enrolled in this study. All cases had a histological diagnosis of endometriosis and received laparoscopic surgery before IVF treatment. Patients who failed to get pregnant in at least the first 6 months after laparoscopic surgery, although they had intercourse at least twice a week, were eligible to receive IVF–embryo transfer (ET) treatment. The results of semen examination of patients’ husbands were within the World Health Organization (WHO) reference range [10]. No couple used any contraception. In addition, the patients did not receive any ovulation stimulation treatment within 6 months before the IVF treatment. The exclusion criteria (determined by laparoscopy, ultrasound examination, and hormonal tests) were uterine myoma, adenomyoma, congenital structural abnormalities of the reproductive tract, pelvic tuberculosis, ovarian tumour, polycystic ovary syndrome, hyperprolactinaemia, adrenal disease, thyroid disease or other endocrine disease, and male infertility.

### r-AFS classification method

According to the standards of r-AFS classification (1985 and 1996) [4,5] the lesion score and total score were calculated based on a retrospective analysis of the surgery report in the hospital medical records. The endometriosis stage of each patient was classified as well. We obtained institutional review board approval for retrieving the laparoscopic surgery records and the IVF data.

### EFI score

**Figure 1:**
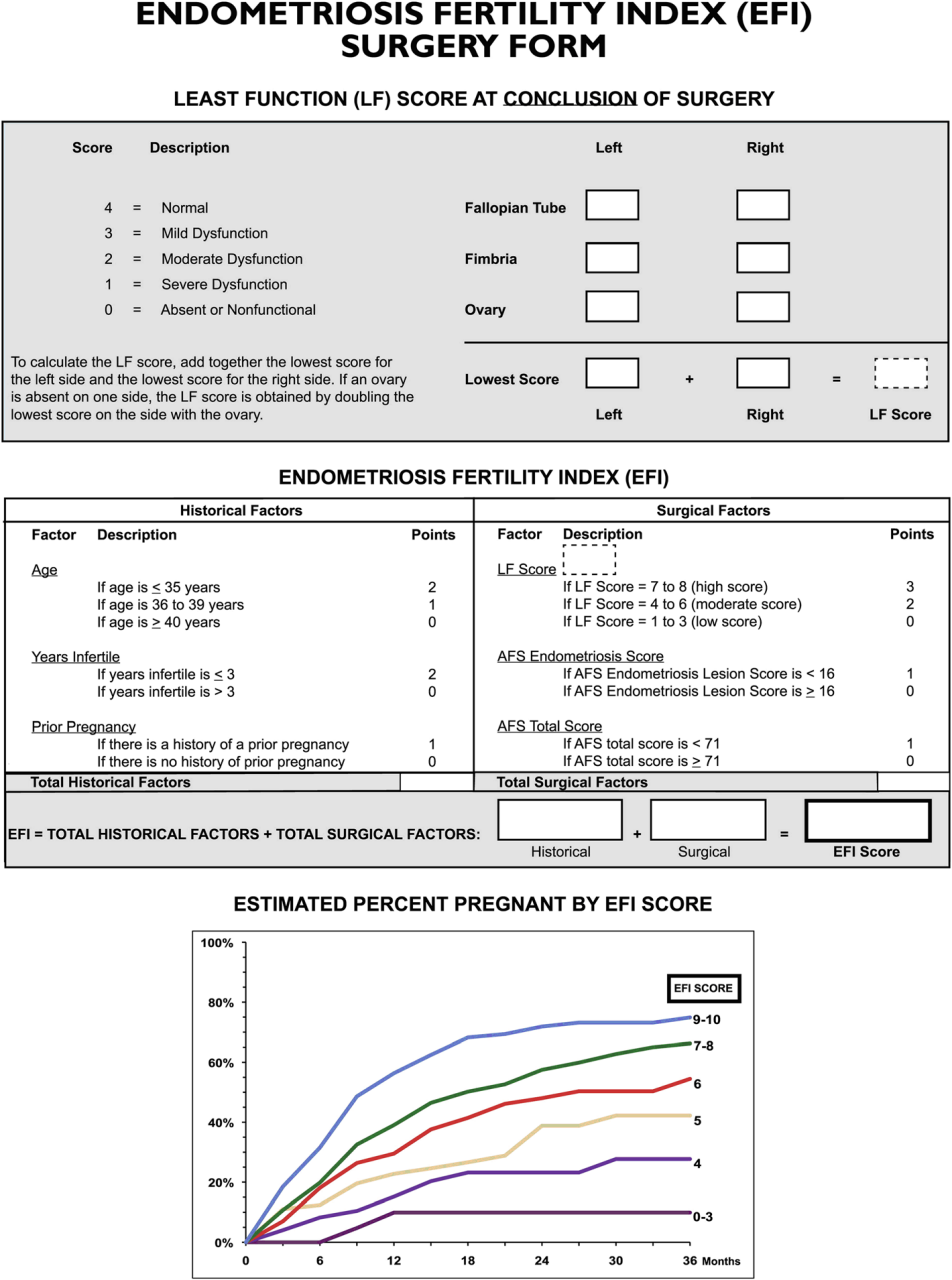
**Rating scale of the least-function score and the Endometriosis Fertility Index.** Note: The Table is from Adamson GD, Pasta DJ. Endometriosis fertility index: the new, validated endometriosis staging system. Fertil Steril. 2010. 94(5): 1609–1615 [8]. Copyright 2010, with permission from Elsevier.

The EFI score was calculated according to the EFI developed by Adamson and Pasta [8]. It includes the following clinical and surgical factors (Figure 1): age, duration of infertility (years), pregnancy history, least-function (LF) score (including fallopian tubes, tubal fimbriae, and ovaries; LF score = the least score of the left side + the least score of the right side; if any ovary was absent, the LF score was obtained by doubling the LF score of the contralateral side), r-AFS score of the lesion, and total r-AFS score. Figure 1 shows Rating scale of the least-function score and the Endometriosis Fertility Index

### Ovarian stimulation, insemination, and IVF treatment

The prolonged gonadotropin-releasing hormone agonist protocol was performed as follows. The patients underwent pituitary down-regulation through an intramuscular injection of 3.75 mg of triptorelin acetate (Diphereline; Ipsen Pharma Biotech. France) [11,12]. Twenty-eight to 35 days later, the patients were given a second injection of 1.25 mg (one third of Diphereline®) of triptorelin acetate. Twenty-one to 28 days later, the patients were given a third injection of 0.93 mg (quarter of one Diphereline®) of triptorelin acetate. Then, 1 week later, recombinant follicle-stimulating hormone (rFSH) was used to stimulate follicle development. The initial dose of rFSH (Gonal-F,75 IU/per amp,Merck Serono, Geneva, Switzerland) was 150–300 IU/day, depending on the antral follicle count (AFC), patient age, and basal serum level of FSH. Follicle development was monitored under transvaginal ultrasound guidance. Recombinant human chorionic gonadotropin (hCG) (Ovidrel, Serono) (250 mg) was administered subcutaneously when two or more follicles ≥18 mm in mean diameter were present. Oocytes were retrieved by puncture of the vaginal sac guided by transvaginal ultrasound 35 to 36 hours after hCG administration [13]. Insemination was performed with 150,000 motile sperms cells/mL, always between 14:00 and 15:00 on the day of oocyte retrieval. Fertilisation was assessed 16 to 18 hours after insemination by searching the oocytes for evidence of pronucleus formation and a second polar body. Each embryo was incubated in a separate droplet of G1 medium (VitroLife®, Sweden ) covered with paraffin oil (VitroLife®, Sweden ).

Embryo transfer was performed on the third day after retrieval, and the luteal phase was supplemented with injection of 40 mg of progesterone/day. Biochemical pregnancy was confirmed by measuring serum β-hCG on the 14th day after transfer. Clinical pregnancy was confirmed by the presence of a gestational sac and foetal heart beat on the 30th to 35th day after transfer.

### Embryo evaluation

Fertilisation and pronuclear morphology were assessed 16–18 hours after insemination. Zygotes were checked for the number, size, and position of the pronuclei (PN) as well as the number and location of the nucleolar precursor bodies within the PN, as described previously by Scott et al [14].

The assessment on days 2 and 3 was performed as follows. Morphologic assessments were always performed at the same time (between 8:00 am and 9:30 am). The embryos were identified as grade I (good quality) if they had either 2–4 blastomeres of equal size and less than 20% fragmentation on day 2 or 7–8 blastomeres of equal size and less than 20% fragmentation on day 3. The embryo transfer was performed on the third day after oocyte collection [15]. Three embryos were transferred in the patients who were 35 years or older, and one or two embryos were transferred in the patients who were younger than 35 years. Clinical pregnancy was determined by the presence of a gestational sac and heart beat by a transvaginal ultrasound performed on the 30^th^ to 35^th^ days after the embryo transfer. The culture medium was G5 series(VitroLife®, Sweden).

### Hormone assays

Blood samples were obtained from each patient with regular menstruation prior to the initiation of a stimulation cycle, at 9:00 am-10:00 am during days 2 to 4 of the menstrual cycle. A Beckman Coulter UniCel DxI 800 and the associated reagents (Beckman Coulter, Los Angeles, USA) were used to determine the serum sex hormone levels (basal FSH, basal luteinising hormone, basal oestradiol) by chemiluminescence.

### Data analysis and statistical methods

Estimation of sample size: To compare the EFI score and r-AFS classification on the fecundity and IVF outcomes, we used a diagnostic test to estimate the sample size. Because the two indices would be used on the same population, we used the EFI score to calculate the sample size. The pregnancy state after IVF was the gold standard, and positive meant pregnancy and negative meant non-pregnancy.

A total of 161 cases were collected from January 2008 to December 2011, who were diagnosed with endometriosis and received IVF treatment after laparoscopy. After the area under the receiver operating characteristic curve (AUC ROC) for EFI score was calculated, the cut-off score was 6. Therefore, the 161 cases were divided into two groups, a ≤5 score group and a ≥6 score group. We next calculated the necessary sample size as the following formula Formula for sample size estimation).

To avoid excessive variability and minimise bias, we paid close attention to the following before calculating the sample size. Sample drawing: the non-pregnant women with endometriosis were enrolled 6 months after laparoscopic surgery if they had requested IVF treatment. The gold standard of the diagnostic test was ‘pregnancy’. We chose an accuracy index α = 0.05, power = 90%, and a bilateral variability test. All the surgeries were performed by three highly experienced gynaecologic surgeons who worked in the same hospital and the assessments of the endometriosis classification staged were also performed by these three surgeons. They had concordant opinions and judgments of endometriosis traits. The scores from their operation records were comparable.

Formula for sample size estimation [16]:

$$\begin{array}{c} \begin{array}{c} \begin{array}{c} n={\left\{\frac{{Z_{1}}_{-a/2}\sqrt{2\bar{p}}+Z_{1-\beta }\sqrt{2\left(p_{1}-p\right)\left(p_{1}-p\right)/\bar{p}}}{p_{1}-p_{2}}\right\}}^{2}\\ p_{1}=\frac{a+b}{N} \end{array}\\ p_{2}=\frac{a+b}{N} \end{array}\\ p=\frac{a}{N}\\ \bar{p}=\frac{p_{1}+p_{2}-2p}{2} \end{array}$$

Data collection: We performed a retrospective analysis of the operation records and the case records from the medical record archive file, medication doses during IVF treatment, number of oocytes retrieved, fertilisation rate, embryo development, and pregnancy rate.

Data calculation: The sample size (n = 196) was determined by the data in Table 1 and above formula for sample size estimation [16,17]. Table 1 shows sample size estimation using the diagnostic test.

**Table 1:** Sample size estimation using the diagnostic test

**EFI score**	**Pregnancy**		**Total**
	**Positive**	**Negative**	
*≥6 score*	a(44)	b(47)	a + b(91)
*≤5 score*	c(23)	d(47)	c + d(70)
*Total*	a + c(67)	b + d(94)	N(161)

After the sample size was determined, the cases were continuously collected until 31 July 2012. A total of 199 cases were enrolled. ROC curves were used to analyse the predictive values of the EFI and r-AFS indices for clinical pregnancy, and their accuracies were evaluated by sensitivity, specificity, and Youden’s index. The ROC analysis was used to calculate the cut-off point of each index for successful prediction. The ROC of the EFI score and the ROC of r-AFS classification were first drawn in SPSS v20.0, and the cut-off point of each diagnosis was calculated. The AUC of the EFI score and the AUC of r-AFS were compared. The cases were divided into two groups according to the EFI score (≤5 and ≥6). The same populations were divided into two groups according to the r-AFS classification (stage I-II and stage III-IV). The goodness of fit of each diagnostic system was assessed by the AUC ROC, in which a higher AUC value indicated a better fit to predict implantation. Significance was defined as a two-tailed *P* value less than 0.05.

The statistical analyses were performed using SPSS v20.0 software. The normally distributed data of quantitative variation and continuous variables are reported as the mean ± standard deviation and were compared using the *t*-test. The non-normally distributed data are reported as the median (inter-quartile range) and were compared using the Mann–Whitney test. Comparison between the two EFI groups and between the two r-AFS groups was performed with the chi-squared test for categorical variables or the *t*-test and Mann–Whitney test for continuous and ordinal variables. All tests were two tailed, and P < 0.05 was considered statistically significant. Whenever appropriate, writing and analysis followed the STARD [18].

## Results

Baseline and cycle characteristics of the 199 patients who were enrolled in the study (Table 2):

**Table 2:** Baseline and cycle characteristics of the patients

**Characteristic**	**Total**	**EFI**				**r-AFS**			
		**≤5 score**	**≥6 score**	***t(z)***	***P ***^**c**^	**I-II stage**	**III-IV stage**	***t(z)***	***P ***^**d**^
No. of cycles	199	84	115			61	138		
Average age(y)^a^	32.0 ± 4.2	33.3 ± 4.9	31.1 ± 3.3	3.789	0.000	32.5 ± 3.7	31.8 ± 4.4	1.085	0.279
Duration of infertility(y)^b^	5.0(3.0–7.0)	6.0(4.0–9.0)	4.0(2.0-6.0)	−3.893	0.000	5.0(3.5–7.0)	4.0(2.0–7.0)	−1.176	0.240
BMI(kg/m^2^)^b^	20.2(18.8–21.7)	20.2 (18.2–22.0)	20.3(18.9–21.7)	−0.374	0.708	20.6(19.1–21.8)	20.0(18.5–21.6)	−1.013	0.311
bFSH(iu/L)^b^	8.1(6.7–9.9)	8.6 (6.9–11.7	8.0(6.6–9.3)	−1.645	0.100	7.9(6.3–9.3)	8.3(6.9–11.0)	−1.683	0.092
bLH(iu/L)^b^	3.8(2.7–5.5)	3.8(2.7–5.3)	3.8(2.9–5.6)	−0.009	0.993	3.7(2.7–5.5)	4.0(2.9–5.5)	−0.706	0.480
E2(ng/L)^b^	42.4(27.0–61.9)	47.4(29.7–57.9)	42.0(27.3–64.7)	−0.957	0.339	42.0(22.9–64.3)	45.9(27.1–60.5)	−0.864	0.388
Antral follicle count^b^	10(6.0–14.0)	8.5(6.0–13.0)	11.0(8.0–14.0)	−2.212	0.027	10.0(8.0–14.0)	9.5(6.0–14.0)	−1.662	0.097
E2 level on D_HCG_ (ng/L)^b^	1987.2(1122.6–3369.1)	1661.3 (925.2–3599.5)	2110.3(1239.8–3334.0)	−1.255	0.209	2352.6 (1331.7–4241.5)	1796.9 (1012.2–3251.3)	−2.065	0.039
Start dose of Gn(iu/day)^b^	225.0 (150.0–300.0)	225.0(150.0–300.0)	225.0(150.0–225.0)	−2.401	0.016	225.0 (150.0–225.0)	225.0 (150.0–300.0)	−1.853	0.064
Duration of stimulation(d)^b^	10(9.0 ~ 12.0)	10(9.0–12.0)	10(9.0–12.0)	−0.497	0.619	11(9–11)	10(9–12)	−0.214	0.830
Total dose of Gn(iu)^a^	2286.6 ± 926.4	2439.3 ± 1076.8	2175.0 ± 785.3	−1.909	0.058	2169.6 ± 784.5	2338.3 ± 980.8	−1.186	0.237

Note: The Shapiro-Wilk test was performed to determine the normality of distribution. P >0.05 indicates the data followed a normal distribution.

^a^The normally distributed data are presented as mean ± SD, including age and total dose of Gn. The *t*-test was performed to analyse statistical significance.

^b^The non-normally distributed data are presented as median (25th-75th percentile). The non-parametric Mann–Whitney test was performed to calculate the Z-score.

^c^Comparison between the two EFI score groups (score ≤5 vs score ≥6).

^d^Comparison between the two r-AFS stage groups (stage I-II vs stage III-IV).

The average age was 32.0 ± 4.2 years. The regular menstruation period was 27 to 33 days and lasted 5 to 7 days. The average body mass index (BMI) was 20.2 kg/m^2^. Fifty-two patients had received ovarian stimulation therapy once before laparoscopic surgery, and 135 had primary infertility and 64 secondary infertility. Five patients with secondary infertility had experienced an ectopic pregnancy, and three of these five cases had a history of elective abortion before ectopic pregnancy. Fifteen patients had once spontaneous miscarriage, and 44 had received an elective abortion. All 199 women had attempted to conceive by natural methods and were unsuccessful after 6 months and so requested IVF treatment. The outcome of IVF was that 85 women achieved pregnancy in the first, fresh cycle, and 107 women achieved pregnancy after a fresh cycle and a freeze-thaw cycle. The cumulative pregnancy rate was 53.8%.

### ROCs for pregnancy diagnosis of the EFI and r-AFS indices

The 199 fresh IVF-ET cycles were analysed in this retrospective study. In fresh IVF-ET cycles, 85 cycles resulted in pregnancy and 114 cycles resulted in non-pregnancy. The EFI showed an AUC = 0.641, Standard Error (SE) = 0.039, P = 0.001, 95% Confidence Interval (CI) = 0.564-0.717, cut-off score = 6 score, sensitivity =71.8%, specificity = 52.6%, and Youden index = 0.244 (Youden index = sensitivity + specificity-1). The r-AFS classification showed an AUC = 0.445, SE = 0.041, P = 0.184, and 95% CI = 0.364-0.526.

As shown in “ROC of pregnancy in fresh IVF cycles for the EFI score and r-AFS classification” subsection, the AUC of the EFI score was larger than that of the r-AFS classification. This suggests that the EFI score had a greater ability to predict IVF pregnancy outcome than r-AFS.

**Figure 2:**
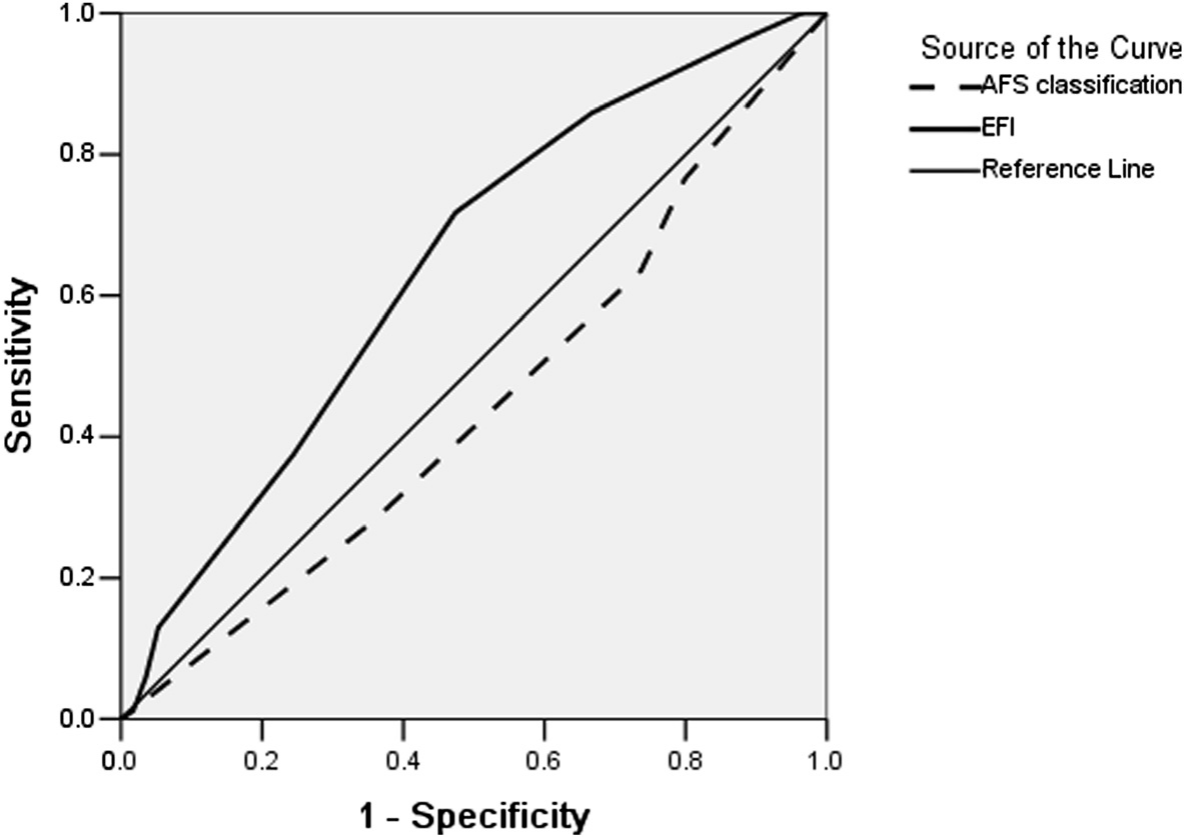
ROC of pregnancy in fresh IVF cycles for the EFI score and r-AFS classification.

Using the cut-off EFI score of 6, we compared each IVF item between the ≤5 EFI score group and the ≥ EFI 6 score group. We performed a similar comparison for each IVF item between the r-AFS stage I-II group and the stage III-IV group. Figure 2 shows ROC of pregnancy in fresh IVF cycles for the EFI score and r-AFS classification.

Comparison between the two EFI score groups and between the two r-AFS stage groups (Tables 2 and 3).

**Table 3:** The embryos quality and outcome of IVF-ET

**Characteristic**		**EFI**				**r-AFS**			
	**Total**	**≤5 score**	**≥6 score**	***z (χ***^***2***^ ***)***	***P***^**c**^	**I-II stage**	**III-IV stage**	**z (χ2)**	***P***^***d***^
No. of cycles	199	84	115			61	138		
No. of oocytes retrieved^a^	8.0(4.0–12.0)	6.0(3.0–10.8)	9.0(5.0–14.0)	−3.071	0.002	9.0(5.0–15.0)	7.0(3.0–11.3)	−2.454	0.014
No. of fertilization	5.0(3.0–9.0)	4.0(2.0–8.0)	6.0(4.0–10.0)	−3.292	0.001	6.0(4.0–10.0)	5.0(2.8–8.0)	−2.493	0.013
No. of fertilization 2PN^a^	4.0(2.0-7.0)	3.5(1.0-7.0)	5.0(3.0–8.0)	−2.735	0.006	5.0(3.0–9.0)	4.0(2.0-7.0)	−1.851	0.064
Rate of 2PN fertilization^b^	1082/1834(59.0%)	414/676(61.2)	668/1158(57.7)	2.232	0.135	379/671(56.5)	703/1163(60.4)	2.764	0.096
Rate of polypronucleus zygote(%)^b^	154/1834(8.4%)	51/676(7.5)	103/1158(8.9)	1.012	0.314	70/671(10.4)	84/1163(7.2)	5.698	0.017
No. of cleavage from 2PN	4.0(2.0–7.0)	3.0(1.0-6.0)	5.0(3.0–7.0)	−2.980	0.003	5.0(3.0–9.0)	4.0(2.0–6.0)	−2.082	0.037
Cleavage rate from 2PN zygote^b^	1045/1236(84.5)	391/465(84.1)	654/771 (84.8)	0.121	0.728	372/449(82.9)	673/787(85.5)	0.696	0.404
Availability of embryos(%)^b^	924/1306(70.8)	347/496(70.0)	577/810(71.2)	0.242	0.623	310/475(65.3)	614/831(73.9)	10.862	0.001
Top quality embryos(%)^b^	139/1045(13.3)	53/391(13.6)	86/654(13.1)	0.035	0.852	52/372(13.9)	87/673(12.9)	0.230	0.632
No. Of embryos transferred	2.0(2.0–2.0)	2.0(1.0–2.75)	2.0(2.0-2.0)	−1.920	0.055	2.0(2.0–3.0)	2.0(2.0–2.0)	−2.349	0.019
Implantation rate(%)^b^	120/386(31.1)	37/148(25.0)	83/238(34.9)	4.153	0.042	43/131(32.8)	77/255(30.2)	0.279	0.597
Clinical pregnancy rate(%)^b^	85/199(42.7)	24/84(28.6)	61/115(53.0)	11.881	0.001	31/61(50.8)	54/138(39.1)	2.362	0.124
Accumulation rate of pregnancy^b^	107/199(53.8)	33/84(39.2)	74/115(64.3)	12.266	0.000	37/61(60.7)	70/138(50.7)	1.678	0.195

Note: Rate of 2PN fertilisation = number of 2PN zygotes/number of oocytes retrieved; rate of polypronucleus zygotes = number of polypronucleus zygotes/number of oocytes retrieved; rate of cleavage from 2PN = number of cleavages from 2PN/number of zygotes; embryo availability rate = (number of frozen embryos + number of ET embryos)/number of cleavage-stage embryos; top-quality embryos = number of top-quality embryos/number of cleavage-stage embryos from 2PN; implantation rate = number of gestational sacs/number of ET embryos; clinical pregnancy rate = number of pregnancy cycles/number of embryo transfer cycles; accumulation rate of pregnancy = number of pregnancy cycles for fresh transferred embryos or freeze-thawed transferred embryos in a single cycle of ovulation stimulation/number of cycles for ovulation stimulation.

Definition of top-quality embryo: seven to eight blastomeres of equal size and less than 10% fragmentation can be seen on day 3 ( Alpha Scientists in Reproductive Medicine and ESHRE Special Interest Group of Embryology, 2011) [19].

^a^The non-normally distributed data are presented as median (25th-75th percentile). The non-parametric Mann–Whitney test was performed to calculate the Z-score.

^b^The chi-squared test was performed to compare rates between groups.

^c^Comparison between the EFI score ≤5 and ≥6 groups. (score ≤5 vs score ≥6).

^d^Comparison between the r-AFS stage I-II and stage III-IV groups (stage I-II vs stage III-IV).

First, baseline and IVF cycle characteristics of the patients in the two EFI groups were compared by the *t*-test and Mann–Whitney test. Then, the medication doses during IVF treatment, embryo development, and IVF outcomes were analysed. Patient age and duration of infertility are included in the EFI score, and the patient age in the ≤5 score group was greater than that in the ≥6 score group. The duration of infertility in the ≤5 score group was longer than that in the ≥6 score group. Among the factors we used to evaluate the ovarian reserve in the EFI score, the AFC in the ≥6 score group was greater than that of the ≤5 score group, and the starting dose and total dose of Gn of the ≥6 score group were less than those of the ≤5 score group. The effectiveness of ovarian stimulation in the ≥6 score group was better than that in the ≤5 score group. The oestradiol level of the day of hCG and the number of oocytes retrieved were greater in the ≥6 score group. The number of two-pronucleus (2PN) fertilisations and the number of cleavages from 2PN were greater in the ≥6 score group. Implantation rate, clinical pregnancy rate, and cumulative pregnancy rate were also higher in the ≥6 score group.

We next compared the various factors between the r-AFS stage I-II and stage III-IV groups. There was no significant difference between groups in the factors that affect ovarian reserve, including basal FSH and AFC. The number of oocytes retrieved and the number of fertilisations were greater in the stage I-II group than in the stage III-IV group. However, the availability of embryos was lower in the stage I-II group. There were no significant differences in implantation rate, clinical pregnancy rate, or accumulation rate of pregnancy between the two groups.

Among the 199 cases, 386 embryos were transferred. There were 53 cycles with singletons, 29 cycles with twin pregnancies, and 3 cycles with triplet pregnancies.

## Discussion

### The relationship between r-AFS classification and pregnancy rate in patients receiving IVF treatment after laparoscopic surgery

The r-AFS classification depends on the results of laparoscopic examination and laparotomy. The staging of endometriosis requires the detailed observation and recording of the site, number, size, and depth of the endometriosis lesions, as well as the degree of adhesions, to define the final score. The r-AFS score is mainly used to assess disease severity and develop a post-operative treatment plan. The diameter of a ‘chocolate cyst’ in the ovary plays a critical role in determining the r-AFS score. For patients with endometriosis who want to become pregnant, this staging method has limited ability to predict future fertility after surgery [8]. Opoien et al. [20]conducted a large retrospective study of r-AFS classification of endometriosis and the success rate of using IVF/intracytoplasmic sperm injection in treating female infertility due to fallopian tube disease. They found that there was no difference in pregnancy rate or childbirth rate between patients with different stages of endometriosis or fallopian tube diseases after patients with adenomyoma were excluded. Similarly, the present study excluded patients with adenomyoma and only evaluated the outcome of IVF in the treatment of infertility due to fallopian tube disease. Our results show that there was no statistical difference between the implantation rate and the pregnancy rate in patients with different stages of endometriosis, consistent with the above study. In addition, the AUC ROC of r-AFS was 0.445, which was not significant for diagnosis. Therefore, our data suggest that the r-AFS classification can predict neither future pregnancy in endometriosis patients nor the outcome of IVF. We speculate that the EFI score maybe more sensitive in predicting pregnancy because it not only considers the size and number of lesions and the degree of local adhesion but also consider other reproductive factors, such as age, infertility duration, or fallopian tube and ovarian function.

### The relationship between EFI score and pregnancy rate in patients receiving IVF treatment after laparoscopic surgery

In 2002, Fujushita et al. modified the AFS classification of endometriosis by adding the TOP score, (fallopian tubes, ovaries, peritoneum, and other factors) [21]. However, they did not consider the patient’s age or other factors affecting pregnancy. Adamson and Pasta (2010) further revised and updated the AFS classification system. They prospectively collected detailed clinical and surgical data of 579 patients with endometriosis and analysed 275 variables associated with pregnancy, thereby establishing the EFI. In addition, they confirmed that the EFI had a close correlation with pregnancy rate in 222 patients. In 2013, Tomassetti C et al. suggested that the EFI could be used clinically to counsel infertile endometriosis patients receiving reproductive surgery in specialized centers about their post-operative conception options [9]. However, their study did not include patients receiving IVF treatment after endometriosis surgery. We believed that in the course of ovarian stimulation for IVF treatment, it would be better to predict fertility after endometriosis surgery by comparing the dosage of medication, number of oocytes retrieved, embryo quality, implantation rate, and pregnancy rate in different groups of EFI scores. Unlike the r-AFS classification, the EFI objectively evaluates factors closely associated with female fertility, such as fallopian tube, tubal fimbria, and ovarian function. It incorporates the LF score which can evaluate the reproductive potential of pelvic organs and comprehensively includes several objective factors, such as patient’s age, duration of infertility, and pregnancy history. According to our data, the AUC ROC for embryo transfer during a fresh IVF cycle was only 0.641 using the EFI score. Patients with a score of 6 accounted for the largest proportion in our study. Patients with a score ≥6 had a significantly higher pregnancy rate than patients with a score ≤5. This is slightly different from the study of Adamson *et al*. According to their study, patients with a score of 7 accounted for the largest proportion [8]. Patients with scores of 8–10 had higher cumulative pregnancy rates than patients with scores of 5–7. A plausible explanation for this difference is that the participants in the two studies used different methods to conceive. The participants in our study were patients receiving IVF treatment after surgery. Therefore, the fallopian tube factors had little impact on the outcome of their IVF treatment, which could have lowered the cut-off point in the EFI in comparison to the patients who planned pregnancy naturally after the surgery.

The EFI incorporates patient age and duration of infertility. As shown in Table 2, no significant differences were observed in the baseline BMI and basal sex hormone levels between patients with EFI scores ≤5 and ≥6. The antral follicle count and the starting dose of Gn were different between the two groups (total Gn dose was also slightly different). This suggests that physicians estimated the starting Gn dose based on BMI, basal sex hormone levels, and antral follicle count in patients with different EFI scores. Therefore, both starting and total Gn doses were affected by oocyte reserve. The number of oocytes retrieved was also relatively higher in the ≥6 score group. However, although the rates of available embryos and top-quality embryos showed no significant differences between groups, the implantation rate and pregnancy rate were higher in the ≥6 score group, suggesting that a score of 6 can be considered an appropriate cut-off point when assessing EFI in endometriosis patients. The pregnancy rate was also higher in patients with score ≥6.

In summary, the EFI incorporates age and duration of infertility; hence, it objectively evaluates the function of reproductive organs better than r-ASF. It has a relatively good predictive power for pregnancy outcomes for patients receiving IVF-ET treatment after laparoscopic surgery. The implantation rate and pregnancy rate were higher in patients with EFI score ≥6 than those with EFI score ≤5. These data provide an important reference to predict the post-operative pregnancy outcome for endometriosis patients, which is the most valuable conclusion of this study.

This study has several limitations. First, there were no significant differences in the rate of available embryos or the rate of top-quality embryos between EFI groups or between r-ASF groups. However, the implantation rate and pregnancy rate were significantly different between groups, suggesting that endometriosis can not only affect the ovaries and quality of oocytes but also the uterine endometrium for embryo implantation. A more convincing support of the implantation environment theory would be if we followed up the development of blastocysts by evaluating day 5 and day 6 blastocysts after implantation. Second, we used pregnancy/non-pregnancy as the relevant outcome to calculate the sample size without considering fertilisation or available embryos. Therefore, as shown in Table 3, the reason the r-AFS stage III-IV group had a low rate of polypronucleus zygotes and a high number of available embryos were that the r-AFS stage I-II group was relatively small. Third, this study failed to demonstrate whether starting an IVF cycle earlier after laparoscopic surgery can improve the specificity and sensitivity of the EFI. Fourth, severe uterine abnormalities, such as uterine fibroids, adversely affect pregnancy. However, the EFI does not incorporate such uterine conditions, a limitation of the scoring system. We acknowledge the impact of uterine conditions on pregnancy and the fact that EFI does not incorporate them. Therefore, to avoid any bias, we only included cases in this study that did not present with significant uterus fibroids as evaluated by laparoscopy and ultrasound. Finally, this was a retrospective study with the calculated cut-off EFI score of 6. Although the included sample size achieved the requirement for diagnostic tests, it will be necessary to conduct randomised, prospective studies with large numbers of patients to validate and evaluate the EFI and its cut-off point. It is better to analyse pregnancy probability in each stage of the EFI with a larger sample size.

## Conclusions

It suggests that the EFI has more predictive power for IVF outcomes in endometriosis patients than the r-AFS classification. The clinical pregnancy rate was higher in patients with EFI ≥6 score than with EFI ≤5 score.

## Competing interests

The authors declare that they have no competing interests.

## Authors' contributions

WJW conceived the idea and design of the project, collected all cases, did the statistical analysis, drafted the manuscript, edited the final document and acquired funding. RQL collected cases. TFF provided access to the statistical analysis and patient data. LLH and NYO collected cases. LAW collected cases and performed laparoscopic surgery. QXZ provided access to the statistical analysis. DZY acquired funding. All authors read and approved the final manuscript.
